# Problematic Social Media Use and Insomnia Severity Among Saudi University Students: Cross-Sectional Indirect-Association Analyses Involving Fatigue and Anxiety

**DOI:** 10.3390/healthcare14203537

**Published:** 2026-10-20

**Authors:** Mohammad Saud Alotaibi

**Affiliations:** Department of Social Work, College of Social Sciences, Umm Al-Qura University, Mecca 24382, Saudi Arabia; msmasoud@uqu.edu.sa

**Keywords:** social media, insomnia, anxiety, fatigue, mental health, university students

## Abstract

Purpose: This study sought to examine cross-sectional associations of problematic social media use with insomnia severity and to estimate indirect associations involving fatigue and anxiety under a hypothesized statistical ordering among Saudi university students. Methods: A cross-sectional online survey of 430 Saudi undergraduate social media users used the Bergen Social Media Addiction Scale, Fatigue Severity Scale, Depression Anxiety Stress Scales anxiety subscale, and Athens Insomnia Scale. Gender and daily social media-use duration were included as covariates in the primary observed-score regression path models; age was examined as a sensitivity covariate. Bootstrapped indirect associations, a paired-bootstrap contrast between two alternative concurrent-mediator specifications, and ordinal confirmatory-factor-analysis sensitivity analyses were conducted. These analyses do not establish temporal order. Results: Problematic social media use was associated with fatigue (B = 0.161, *p* < 0.001), anxiety (B = 0.388, *p* < 0.001), and insomnia severity (B = 0.161, *p* < 0.001) after adjustment. In the hypothesized fatigue-first decomposition, the fatigue-to-anxiety indirect association was B = 0.072, 95% CI [0.045, 0.105]. Its paired numerical difference from the reverse-order decomposition was B = 0.045, 95% CI [0.013, 0.083]. The AIS two-factor measurement sensitivity model fit better than the one-factor model. Daily social media-use duration was not robust across sensitivity analyses. Conclusions: Problematic social media use, fatigue, anxiety, and insomnia severity were concurrently associated in this sample. The indirect-effect decompositions are statistical descriptions of cross-sectional covariation, not evidence of a temporal, causal, or clinical sequence; longitudinal and experimental studies are needed.

## 1. Introduction

Social media platforms have become deeply embedded in the daily routines of university students worldwide, serving at once as tools for communication, study, and entertainment [1]. This integration is especially pronounced in Saudi Arabia. National survey data indicate that 99.0% of individuals aged 15–74 use the internet, of whom 96.8% go online at least once daily, with mobile phones serving as the primary access device for 98.4% of users [2]. Participation in social networking is the most common online activity in the country, reported by 96.2% of internet users (97.1% of females and 95.7% of males), reflecting the centrality of social media to daily life [2].

While moderate use has clear benefits for learning and social connection, the rising share of students whose use becomes compulsive has raised real clinical and public health concerns.

### 1.1. Problematic Social Media Use

Problematic social media use describes a pattern of excessive, poorly controlled engagement in which the crowding out of other life domains, rather than time spent alone, gradually interferes with daily functioning across social, academic, and relational spheres [3]. The literature has labeled this phenomenon in several overlapping ways, including “social media addiction,” “problematic social media use,” “social networking site addiction,” and “compulsive social media use,” among others, terms frequently used interchangeably to describe the same underlying behavior [4]. Notably, this condition is not yet formally recognized in major diagnostic systems such as the *International Classification of Diseases* (ICD-11) and the *Diagnostic and Statistical Manual of Mental Disorders* (DSM-5) [5]. The present study uses the broader term “problematic social media use,” while drawing on the behavioral addiction framework behind its most widely used measures. This framework conceptualizes problematic use through the same core components that define substance-related and behavioral addictions, such as salience, withdrawal, and conflict [6]. A recent global meta-analysis reported a pooled prevalence of social media addiction among university students of 18.4%, with Asia recording the highest rate at 22.8% [7]. Among Saudi university students, however, rates have reached 55.2% in a single-university sample of medical students [8], a figure corroborated by a subsequent multi-university Saudi sample of 978 students across three public universities, of whom 52.8% met BSMAS addiction criteria [9]. A systematic review with meta-analyses of 98 studies (*N* = 102,683) found that problematic social media use was associated with depression, anxiety, poorer wellbeing, and sleep problems, with sleep disruption linked specifically to problematic use rather than to general social media use [10].

### 1.2. Fatigue

Fatigue is defined as the subjective awareness of a decreased capacity for physical or mental activity resulting from an imbalance in the availability, use, or restoration of the resources required for that activity [11]. The clearest evidence that social media addiction contributes to fatigue among university students comes from a large sample of 2744 students, where addiction increased the odds of social media fatigue nearly fourfold [12]. Network analysis points to a similar pattern, showing that heavy use is associated with substantial reductions in mental resources and greater exhaustion when facing tasks that demand concentration [13]. This link also extends to academic outcomes: a serial mediation study found that fatigue accounted for a larger share of the effect of social media addiction on academic engagement than sleep quality did [14].

### 1.3. Anxiety

Anxiety is defined as a future-oriented mood state involving heightened threat appraisal and a coordinated cognitive, affective, physiological, and behavioral response system that prepares the individual for anticipated danger [15]. A meta-analysis of studies among students found that social media addiction is positively and consistently associated with anxiety, alongside depression and loneliness [16]. This pattern also appears in Saudi samples, where students with anxiety symptoms scored significantly higher on measures of social media addiction [8]. The relationship between fatigue and anxiety, however, appears to run in both directions. A longitudinal study of university students found that anxiety can predict later fatigue [17], though in a model where anger, not anxiety, was the primary sequential trigger and the stronger direct predictor of later fatigue, whereas separate longitudinal evidence in a digital context found that fatigue can give rise to anxiety over time [18], based on a narrow, digital-interaction-specific measure of anxiety, a pattern also observed in a broader adult population followed across pandemic lockdown periods [19].

### 1.4. Insomnia Severity

Insomnia is defined as persistent difficulty initiating or maintaining sleep despite adequate opportunity, together with associated daytime impairment [20]. It is strikingly common among university students: a recent meta-analysis found that nearly half experience insomnia symptoms, a rate almost three times higher than in the general population [21]. Evidence linking social media addiction to insomnia has generally supported a positive association: a recent meta-analysis of 40 studies (*N* = 34,441) confirmed an overall relationship between problematic social media use and poorer sleep quality, although effect sizes have varied across individual studies [22]. This pattern has been corroborated within Saudi university samples specifically: a large multi-university study using the same social media addiction measure adopted here likewise reported a significant positive association between social media addiction and insomnia [23]. One pathway that may help explain this link runs through anxiety: cognitive and emotional arousal, including worry and rumination, delays the onset of sleep and sustains wakefulness, a core assumption of the hyperarousal account of insomnia [24]. Within Saudi university samples specifically, insomnia has been shown to function as an active mechanism, mediating the relationship between social media addiction and poorer mental health [23].

### 1.5. Conceptual Framework and Study Model

The framework therefore provides a rationale for examining associations among problematic social media use, fatigue, anxiety, and insomnia severity; it does not test a biological or temporal sequence. The FSS captures perceived fatigue severity and functional interference rather than direct physiological hyperarousal, and the DASS-21 anxiety subscale captures general anxiety rather than pre-sleep cognitive arousal. Accordingly, competing explanations—including bidirectional relationships, sleep-related health factors, caffeine use, medication, mood symptoms, and unmeasured stressors—remain plausible in these cross-sectional data.

Drawing on university-student evidence linking social-media addiction with social-media fatigue and Zoom fatigue with subsequent interaction anxiety [12,18], the fatigue-first ordering was selected as the primary theory-informed statistical specification, with anxiety-first retained as an alternative parameterization given the potential reciprocity between fatigue and anxiety.

The study’s contribution is exploratory: it jointly examines these constructs in a Saudi undergraduate sample and reports explicitly contrasted, alternative cross-sectional regression decompositions. The hypotheses specify theory-informed expectations for the concurrent associations; they are not tests of temporal or causal sequence. The contrast is informative about the fitted data under the two specifications but does not identify mediator order, mechanism, or incremental causal novelty.

Two further factors are taken into account. As Khan et al. 2024 [25] report, based on adolescent samples spanning 40 countries, sex differences shape both social media use and sensitivity to sleep problems, with females more prone to difficulty falling asleep in this context; gender is therefore included as a covariate. This pattern is corroborated within Saudi university populations specifically, where a systematic review and meta-analysis likewise reported higher insomnia prevalence among female students [26]. Daily social media use is also included, since screen time and frequency of use relate to sleep disturbance on their own, and these links hold even when problematic use is measured alongside them [27,28,29].

Saudi undergraduate students were selected as the target population given the widespread use of social media in Saudi Arabia and previous evidence of problematic use and sleep difficulties among university students [8,23,26].

On this basis, the present study tests the following hypotheses:

**H1.**  *Problematic social media use is positively associated with fatigue, anxiety symptoms, and insomnia severity.*

**H2.**  *Fatigue and anxiety will statistically account for part of the conditional association between problematic social media use and insomnia severity.*

**H3.**  *Problematic social media use is indirectly associated with insomnia severity through the serial statistical pathway of fatigue and anxiety symptoms.*

**H4.**  *Daily social media use is expected to be positively associated with insomnia severity beyond the effect of problematic social media use, whereas gender is associated with differences in mean levels of anxiety symptoms and insomnia severity within the adjusted model.*

## 2. Materials and Methods

### 2.1. Participants and Procedure

This cross-sectional study examined concurrent associations among problematic social media use, fatigue, anxiety, and insomnia severity in Saudi university students. Participants were recruited online through university-related and social media channels and completed an anonymous Arabic-language survey. They provided electronic consent before participation. Of 439 respondents who accessed the survey, 9 declined participation; 430 cases were retained for analysis after data screening.

### 2.2. Measures

Demographic information was obtained using a structured questionnaire assessing gender, age, and daily social media use. Social media use was categorized on a five-point ordinal scale ranging from 1 (≤1 h per day) to 5 (>7 h per day).

#### 2.2.1. Problematic Social Media Use

The Bergen Social Media Addiction Scale (BSMAS) was used to assess problematic social media use. It was developed by Andreassen et al. 2016 [30] and consists of six items rated on a five-point scale ranging from 1 (Never) to 5 (Very Often), reflecting patterns of social media use over the past year. Total scores range from 6 to 30, with higher scores indicating greater problematic use. The Arabic version validated by Abiddine et al. 2025 [31] was used in the present study, as validated in Arabic-speaking populations. A subsequent multi-university Saudi sample similarly reported a comparably high addiction prevalence [9]. Internal consistency in the present sample was acceptable (Cronbach’s α = 0.767; McDonald’s ω = 0.773).

#### 2.2.2. Fatigue Severity

The Fatigue Severity Scale—7-item version (FSS-7) was used to assess the functional impact of fatigue on daily activities. The scale was originally developed by Krupp et al. 1989 [32] as a nine-item instrument. The Arabic wording of the retained items was unchanged from the validated Arabic FSS-9 [33], and the administered form comprised seven items corresponding to original FSS items 3–9. Items 1 (“My motivation is lower when I am fatigued”) and 2 (“Exercise brings on my fatigue”) were omitted to reduce respondent burden; this item selection was supported by Rasch analyses [34,35]. Items are rated from 1 (Strongly Disagree) to 7 (Strongly Agree) based on experiences over the past week. The scale score is expressed as the mean of all seven items (range: 1–7), with higher scores reflecting greater fatigue severity. Internal consistency in the present sample was excellent (Cronbach’s α = 0.927; McDonald’s ω = 0.927).

#### 2.2.3. Anxiety Symptoms

Anxiety symptoms were assessed using the anxiety subscale of the Depression Anxiety Stress Scales–21 (DASS-21; [36]). The DASS-21 consists of three seven-item subscales; only the anxiety subscale was used in the present study. This subscale assesses physiological arousal, situational anxiety, and subjective feelings of fear and tension over the previous week. Items are rated from 0 (did not apply to me at all) to 3 (applied to me very much, or most of the time), with higher scores indicating greater anxiety severity. The Arabic version validated by [37] was used. Previous validation in Arabic-speaking populations has demonstrated satisfactory reliability and validity [38]. In the present sample, the anxiety subscale showed good internal consistency (Cronbach’s α = 0.878; McDonald’s ω = 0.879).

#### 2.2.4. Insomnia Severity

Insomnia symptoms were assessed using the Athens Insomnia Scale (AIS; [39]). The AIS is an eight-item self-report measure based on ICD-10 criteria for non-organic insomnia, assessing nighttime sleep difficulties and daytime dysfunction. Participants were instructed to report difficulties that had occurred at least three times per week during the previous month [39]. Items are rated from 0 (no problem) to 3 (severe problem), with total scores ranging from 0 to 24; higher scores indicate greater insomnia severity. The Arabic version adapted by [40] was used in the present study. The scale has demonstrated acceptable construct validity in Arabic-speaking populations, including a Saudi validation supporting a two-factor structure [41]. Internal consistency in the present sample was good (Cronbach’s α = 0.844; McDonald’s ω = 0.846).

### 2.3. Data Collection

Data were collected through an online self-administered questionnaire developed in Arabic and administered via Microsoft Forms (Microsoft Corporation, Redmond, WA, USA) during the spring semester of the 2025–2026 academic year (April–May 2026). The survey link was distributed through official Umm Al-Qura University email communications and informally through social media groups (e.g., Telegram) to facilitate participation among students. Prior to accessing the questionnaire, prospective participants were presented with a detailed information sheet outlining the study objectives, the voluntary nature of participation, the complete anonymity of all responses, and the intended academic use of the data. Electronic informed consent was obtained before survey access was granted; selecting “I agree to participate” constituted a binding electronic consent signature. Participants were explicitly informed of their unconditional right to withdraw at any point without consequence. No personally identifiable information was collected, and full confidentiality was maintained throughout all stages of the study. Completion of the questionnaire required approximately 10 min.

### 2.4. Ethical Approval

This study was conducted in accordance with the ethical protocols approved by the Biomedical Research Ethics Committee at Umm Al-Qura University, Saudi Arabia (IRB Ref: HAPO-02-K-012-2026-03-3284, approved 26 February 2026). All procedures adhered to the ethical standards established by the institutional review board and were consistent with the principles of the Declaration of Helsinki.

### 2.5. Construct Validity and Distinctiveness

Ordinal-item CFAs with WLSMV estimation were conducted as measurement sensitivity analyses. The AIS two-factor solution (nighttime sleep and daytime functioning) fit better than a one-factor solution (scaled difference test *p* < 0.001). A five-factor model separating BSMAS, FSS, DASS-A, AIS nighttime, and AIS daytime indicators fit well (CFI = 0.996, TLI = 0.996, RMSEA = 0.036, SRMR = 0.048). AVE was adequate for FSS, DASS-A, and both AIS factors but was 0.419 for BSMAS; thus, discriminant-validity claims are deliberately limited. These fit indices do not establish cultural/content validity or eliminate possible item-content overlap between fatigue and insomnia measures.

### 2.6. Common-Method and Selection Considerations

A Harman single-factor check found that the first unrotated factor accounted for 34.57% of variance. This is only a limited descriptive diagnostic: it does not rule out common-method bias, especially in a self-report, single-wave online survey. HTMT indices were also descriptive and cannot eliminate shared wording or method effects. Online social-media recruitment may additionally have selected respondents with particular usage or sleep characteristics; therefore, results should not be generalized to all Saudi university students without replication.

### 2.7. Data Analyses

All statistical analyses were conducted using IBM SPSS Statistics for Windows, Version 27.0 (IBM Corp., Armonk, NY, USA) [42], supplemented by the PROCESS macro version 4.2 [43]. Reliability analyses and confirmatory factor analyses were conducted in jamovi Version 2.7 (The jamovi Project, Sydney, Australia) [44].

Regression assumptions were screened for the observed-score equations using distributional summaries, standardized residuals, collinearity, influence diagnostics, and Mahalanobis distance. No case was removed from the primary analyses. Auxiliary Lagrange-multiplier tests for heteroscedasticity and RESET-style tests for functional-form misspecification were also conducted, and robust covariance estimates were examined as a sensitivity analysis; results are reported in the Results section. Because indirect associations are products of coefficients and their sampling distributions are typically asymmetric, inference for these estimates relied on percentile bootstrap confidence intervals based on 5000 resamples rather than normal-theory tests, which reduces reliance on normality assumptions but does not correct for model misspecification or omitted variables.

#### 2.7.1. Preliminary Screening

Missing data were examined across all study variables; no missing values were identified (*N* = 430). Four observations exceeded the raw AIS total-score z-score screening threshold (|z| > 3.29). These observations were retained in the primary analyses. A separate Mahalanobis-distance screen identified 0, 1, and 2 cases in the FSS, DASS, and AIS conditional models, respectively; the DASS-flagged case was also one of the two AIS-flagged cases, yielding two unique Mahalanobis cases. The two Mahalanobis cases did not overlap with the four raw-AIS z-score cases. Sensitivity analyses excluding the four raw-AIS cases (*N* = 426) and, separately, the two Mahalanobis cases (*N* = 428)were conducted. These checks assess numerical robustness, not causal mediation.

#### 2.7.2. Covariate Selection

Gender and daily social media-use duration were included as covariates in the primary observed-score regression models. Age was examined in an age-adjusted sensitivity analysis because of the restricted variability of this undergraduate sample. Daily duration was coded continuously in the primary analyses to represent the ordered response levels and to estimate a linear trend, while a separate sensitivity analysis modeled the five response categories as a categorical factor without assuming equal spacing between categories.

#### 2.7.3. Descriptive and Correlational Analyses

Means, standard deviations, and bivariate Pearson correlations were computed for all continuous study variables to characterize the sample and examine preliminary associations among the constructs of interest.

#### 2.7.4. Primary Analysis: Cross-Sectional Indirect-Association Models

Observed-score regression path models estimated conditional associations while adjusting for gender and daily social media-use duration. Age-adjusted models were estimated as a sensitivity analysis. The term indirect association is used to describe the product-of-coefficients estimates. The fatigue-first model was specified as the hypothesized statistical ordering; the anxiety-first model was estimated as an alternative conditional regression parameterization. A paired 5000-resample percentile-bootstrap contrast compared the two numerical serial decompositions. Neither the models nor their difference identifies temporal precedence, causal mediation, or a clinical sequence.

#### 2.7.5. Effect Size Estimation

Effect sizes were reported at the equation level as f^2^ = R^2^/(1 − R^2^), alongside R^2^ and adjusted R^2^. This quantity describes overall explained variance for each regression equation and is not a unique effect size for any individual path. Unstandardized B coefficients, standardized beta coefficients, and 95% confidence intervals are reported in the tables.

#### 2.7.6. Multicollinearity

Multicollinearity among predictors was assessed using variance inflation factors (VIF); all values were below 2 (range: 1.010–1.538; corresponding tolerance range: 0.650–0.990), indicating no problematic multicollinearity [45]. The use of percentile bootstrap confidence intervals reduces reliance on normality assumptions for indirect effect estimation [43,46].

#### 2.7.7. Measurement and Robustness Sensitivity Analyses

Ordinal CFAs used WLSMV estimation for ordinal items to examine the AIS one- versus two-factor structure and a five-factor measurement model. Additional sensitivity analyses removed four extreme AIS cases (|z| > 3.29), used only AIS nighttime-sleep items (items 1–5), and modeled daily social media-use duration as a categorical factor. These analyses assess robustness of concurrent associations; they do not validate temporal mediation.

## 3. Results

### 3.1. Preliminary Analyses

As shown in Table 1, problematic social media use (BSMAS; M = 17.14, SD = 4.86) was positively associated with fatigue (r = 0.462), anxiety symptoms (r = 0.402), and insomnia (r = 0.435; all *p* < 0.001). Associations were also observed between fatigue and both anxiety symptoms (r = 0.523) and insomnia (r = 0.476; all *p* < 0.001), with the highest correlation between anxiety symptoms and insomnia (r = 0.557, *p* < 0.001).

### 3.2. Primary Cross-Sectional Indirect-Association Analysis

The cross-sectional regression model indicated that problematic social media use showed both a direct association and several indirect pathways with insomnia severity through fatigue and anxiety symptoms. Full results are presented in Table 2.

#### 3.2.1. Direct Associations

In the primary specification (adjusted for gender and daily social media-use duration), problematic social media use was associated with fatigue (B = 0.161, β = 0.460, *p* < 0.001), anxiety symptoms (B = 0.388, β = 0.192, *p* < 0.001), and insomnia severity (B = 0.161, β = 0.168, *p* < 0.001). Fatigue was associated with anxiety symptoms (B = 2.423, β = 0.419, *p* < 0.001), and both fatigue (B = 0.527, β = 0.191, *p* < 0.001) and anxiety symptoms (B = 0.184, β = 0.387, *p* < 0.001) were associated with insomnia severity. The equation-level R^2^ values were 0.219 for fatigue, 0.318 for anxiety symptoms, and 0.398 for insomnia severity. Age-adjusted estimates are reported only as a sensitivity analysis.

Diagnostic checks identified no problematic collinearity (VIF range 1.010–1.538; tolerance range 0.650–0.990) or highly influential observations (maximum Cook’s distance = 0.069; maximum leverage = 0.055; maximum Mahalanobis distance = 23.598). Auxiliary heteroscedasticity tests were not significant for the fatigue equation (*p* = 0.329) but indicated possible heteroscedasticity in the anxiety and insomnia equations (*p* = 0.030 and *p* = 0.021, respectively). RESET-style tests indicated no functional-form concern for the fatigue equation, F(2, 424) = 0.356, *p* = 0.701, but suggested possible functional-form concerns for the anxiety equation, F(2, 423) = 5.220, *p* = 0.006, and the insomnia equation, F(2, 422) = 5.093, *p* = 0.007. Robust covariance estimates preserved the direction and approximate magnitude of the principal associations.

#### 3.2.2. Covariate Associations

Age was not independently associated with fatigue, anxiety symptoms, or insomnia severity in the adjusted models (all ps ≥ 0.08). Gender was associated with the anxiety and insomnia regression equations. Daily social media-use duration showed a borderline association with insomnia severity in the ordinary least-squares model (B = 0.362, *p* = 0.049). However, this association was not robust in the robust-covariance analysis (*p* = 0.056), when daily duration was modeled as five response categories (omnibus *p* = 0.302), or after exclusion of the two Mahalanobis cases (B = 0.316, *p* = 0.089).

#### 3.2.3. Indirect Associations and Ordering Contrast

Under the hypothesized fatigue-first cross-sectional decomposition, the bootstrapped serial indirect association was B = 0.072, 95% CI [0.045, 0.105]. In the reverse conditional ordering, the corresponding estimate was B = 0.028, 95% CI [0.013, 0.046]. Their paired bootstrap difference was B = 0.045, 95% CI [0.013, 0.083]. This difference is reported as a comparison of fitted numerical decompositions, not evidence that fatigue temporally precedes anxiety.

### 3.3. Alternative Cross-Sectional Conditional Ordering

Table 3 presents the alternative cross-sectional conditional ordering in which anxiety symptoms are specified before fatigue (BSMAS → DASS-A → FSS → AIS). The direct associations in this alternative parameterization were positive and statistically significant, including the associations of problematic social media use with anxiety symptoms and fatigue, and the associations of anxiety symptoms and fatigue with insomnia severity. The serial indirect association through anxiety symptoms and fatigue was B = 0.028, 95% CI [0.013, 0.046], compared with B = 0.072, 95% CI [0.045, 0.105], under the fatigue-first specification. The paired difference between the two decompositions was therefore interpreted as a comparison of fitted cross-sectional parameterizations, not as evidence of temporal precedence, causal mediation, or a unique mediator sequence.

**Figure 1:**
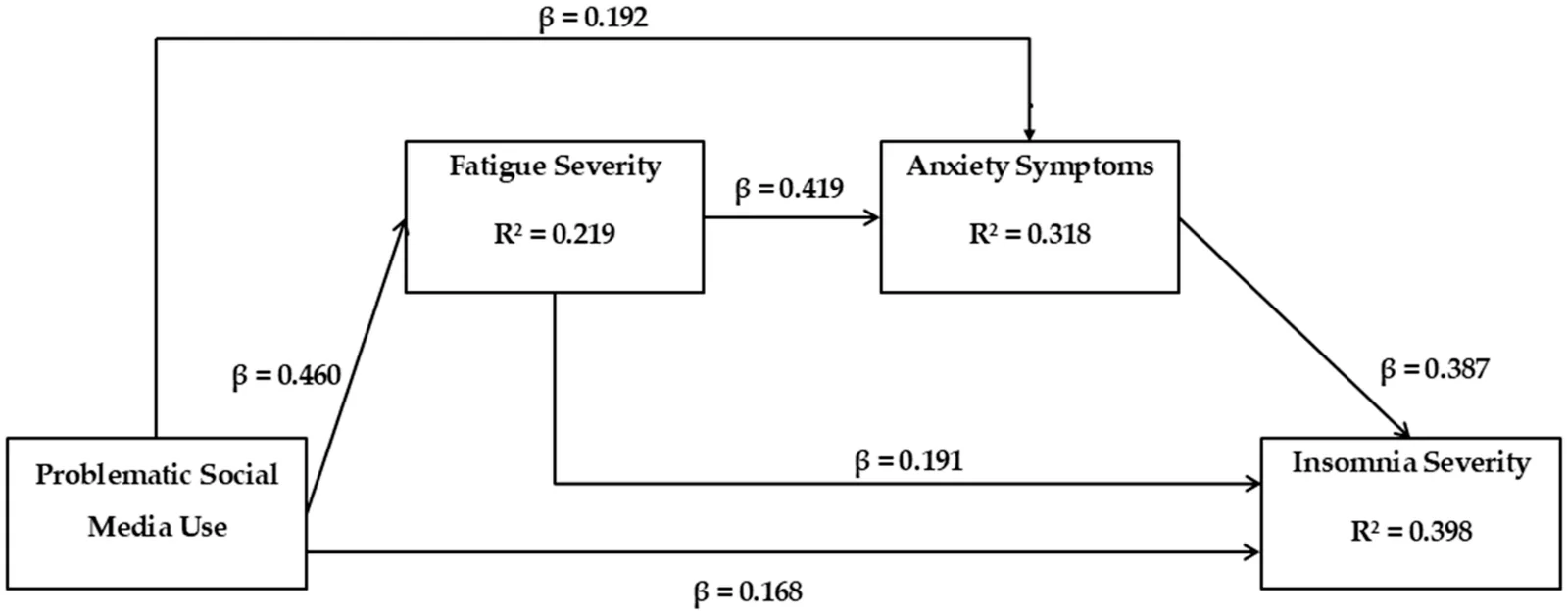
Cross-sectional regression specification linking problematic social media use, fatigue, anxiety, and insomnia severity. Note. Values are standardized regression coefficients (β) and equation-level R^2^ values. Gender and daily social media-use duration were included as covariates in all equations but are not shown. The arrows represent directional regression terms within the fitted cross-sectional specification and do not establish temporal precedence, causal mediation, or a unique psychological sequence. The fatigue-first arrangement reflects the primary, theory-informed statistical specification; the anxiety-first ordering was examined separately as an alternative conditional regression parameterization. The BSMAS–AIS path (β = 0.168) represents the conditional direct association, not the total association.

Figure 1 presents the fitted cross-sectional regression specification.

### 3.4. Measurement and Robustness Sensitivity Analyses

#### 3.4.1. Ordinal Measurement Sensitivity Analysis

The AIS two-factor model fit better than the one-factor model (scaled difference test = 52.21, df = 1, *p* < 0.001). The one-factor model showed CFI = 0.982, TLI = 0.975, RMSEA = 0.095, and SRMR = 0.065, whereas the two-factor model showed CFI = 0.995, TLI = 0.993, RMSEA = 0.051, and SRMR = 0.042. The five-factor model showed CFI = 0.996, TLI = 0.996, RMSEA = 0.036, and SRMR = 0.048. These results support reporting AIS nighttime and daytime content separately in sensitivity analyses, not as a causal chain among the constructs.

#### 3.4.2. Robustness Sensitivity Analysis

After excluding the four observations identified by the raw AIS total-score z-screen (*N* = 426), the fatigue-first serial indirect association was B = 0.0606, 95% bootstrap CI [0.0372, 0.0909], and the reverse-order serial indirect association was B = 0.0258, 95% bootstrap CI [0.0115, 0.0423]. In a separate sensitivity analysis excluding the two unique Mahalanobis cases (*N* = 428), the corresponding fatigue-first and reverse-order serial indirect associations were B = 0.0755, 95% bootstrap CI [0.0472, 0.1111], and B = 0.0287, 95% bootstrap CI [0.0125, 0.0477], respectively. These analyses assess numerical robustness rather than causal mediation. When insomnia severity was restricted to AIS nighttime items, problematic social media use, fatigue, and anxiety remained concurrently associated with the outcome (all *p* ≤ 0.001).

## 4. Discussion

This study found concurrent associations of problematic social media use with fatigue, anxiety, and insomnia severity among Saudi university students. The results are compatible with several theoretical accounts, but the single-wave self-report design cannot determine whether any construct precedes, causes, or mediates another.

In line with H1, problematic social media use was positively and significantly associated with fatigue (B = 0.161, β = 0.460), anxiety (B = 0.388, β = 0.192), and insomnia severity (B = 0.161, β = 0.168; all *p* < 0.001). Each of these associations aligns with patterns reported in recent student samples. The link with fatigue is consistent with Zhu et al. 2023 [12], who found that higher social media addiction corresponded to a markedly greater likelihood of social media fatigue among 2744 university students (OR = 3.775, 95% CI [3.141, 4.537]), and with Feng et al. 2025 [13], whose network analysis linked problematic short-video use with academic burnout and fatigue-related symptoms. The association with anxiety echoes the meta-analytic findings of Jing et al. 2025 [16], who reported a pooled correlation of 0.31 (95% CI [0.25, 0.36]) across nine studies involving 8839 students, as well as Saudi evidence that students with higher anxiety score higher on measures of problematic use [8]. The association with insomnia corresponds with Jameel et al. 2025 [23], who reported a comparable link between social media addiction and insomnia (r = 0.43, *p* < 0.01) among 727 students from three Saudi universities; although the present study remains single-institutional, this consistency suggests that the association is not confined to one Saudi university setting. These comparisons indicate consistency in the direction of associations, although differences in constructs, measures, and statistical models limit direct comparison of their magnitudes.

Recent longitudinal research distinguishes problematic social media use from general screen exposure by focusing on compulsive engagement and sleep-specific outcomes. Ahmed et al. 2025 [48] found that insomnia symptoms were a stronger mediator than global sleep quality in the association between problematic social media use and later mental-health symptoms among young adults in Bangladesh. In contrast, recent Saudi research examined overall screen time and pre-bedtime exposure rather than problematic social media use specifically [49]. Together, these findings suggest that both exposure quantity and problematic engagement may be relevant, while the present study adds cross-sectional evidence using a validated problematic-use measure in Saudi university students.

What the present study adds is the joint examination of these concurrent associations in one model. Earlier Saudi work has often assessed these variables in isolation or in pairwise analyses. Their co-occurrence supports future prospective hypothesis testing but does not establish any pathway or sequence.

Consistent with H2, fatigue and anxiety symptoms statistically accounted for part of the conditional association between problematic social media use and insomnia severity. This finding is broadly consistent with Zhuang et al. 2023 [14], who reported separate and serial indirect associations involving sleep quality and fatigue between social media addiction and academic engagement in a cross-sectional study, although their outcome and mediator specification differed from those examined here. Consistent with H3, the hypothesized fatigue-first serial indirect association was positive (B = 0.072, 95% CI [0.045, 0.105]). Evidence relevant to the fatigue–anxiety component comes from Buyukcebeci 2025 [18], who reported a small positive longitudinal association between Zoom fatigue and subsequent interaction anxiety (β = 0.084, *p* < 0.05); however, the constructs differed from those measured here, and the study did not examine the complete pathway proposed in H3. The anxiety-first specification was examined separately as a transparency and robustness analysis. Its estimate was B = 0.028 (95% CI [0.013, 0.046]), with a paired numerical contrast of B = 0.045 (95% CI [0.013, 0.083]). This contrast reflects sensitivity to alternative cross-sectional regression decompositions and does not establish temporal precedence, causal mediation, or a unique psychological sequence.

The fatigue and anxiety product-of-coefficients estimates should be read as statistical decompositions of cross-sectional covariation. Although the fatigue-first decomposition was numerically larger than the reverse-order decomposition, the paired contrast does not adjudicate temporal sequence. Fatigue may be a consequence of insomnia, anxiety may precede or follow sleep disruption, and unmeasured common causes may generate the observed pattern.

The alternative-ordering analysis is therefore presented as a transparency and robustness exercise rather than evidence for a unique serial mechanism. Longitudinal designs with repeated measures, experimental manipulation, or intensive daily assessment are needed before drawing claims about directionality.

Theoretical interpretation should remain construct-specific. FSS scores describe perceived fatigue and functional interference, and DASS-A scores describe general anxiety; neither directly measures physiological hyperarousal or pre-sleep cognitive arousal. Future work should include measures of sleep timing, arousal, health conditions, stimulant use, medication, mood symptoms, and objective or prospective sleep indicators.

The present findings may nevertheless guide hypothesis generation for culturally responsive prospective research. Any practical implication is limited to screening and awareness, not to treating the fitted cross-sectional ordering as a clinical intervention sequence.

Daily use duration should also be interpreted cautiously. The association also weakened under robust covariance estimation (*p* = 0.056), reinforcing that its significance was not stable across specifications. Its borderline linear association with insomnia severity disappeared in the categorical-duration sensitivity analysis, and the lowest duration category was small (*n* = 9). This pattern does not support a stable independent duration effect in the present data.

The anxiety and insomnia equations showed possible heteroscedasticity and functional-form concerns, whereas the fatigue equation did not. Robust covariance estimates nonetheless preserved the principal associations. These results are interpreted as adjusted cross-sectional associations, not as evidence of causal mediation or a verified temporal chain.

Regarding H4, daily use duration showed only a borderline linear association with insomnia severity (B = 0.362, *p* = 0.049), which was not supported by the categorical-duration omnibus test (F(4, 420) = 1.219, *p* = 0.302); gender, by contrast, was independently associated with both anxiety and insomnia severity in the adjusted model. Regarding gender, female participants reported significantly higher anxiety but significantly lower insomnia severity than male participants, independent of problematic social media use, fatigue, and daily use duration. Because gender was modeled as a covariate rather than a moderator, this indicates a difference in average levels of anxiety and insomnia between men and women, not a difference in how the two relate to one another. The lower insomnia severity among female students is notable given that prior evidence, including work cited earlier in this study, has generally linked female gender to greater vulnerability to sleep difficulties, albeit primarily in adolescent and non-Saudi samples [25]. This divergence may simply reflect sample-specific variation, and it is offered here as an observation rather than an explanation, since testing why anxiety and insomnia diverge by gender would require treating gender as a moderator, which the present design does not support.

## 5. Limitations

Several limitations are central. First, the cross-sectional design precludes temporal, causal, and mediation claims. Second, all measures were self-reported in a single survey, leaving common-method, recall, and shared-item-content bias possible. Third, online recruitment through a convenience sample from a single institution may have introduced selection bias, and the survey administration record was not available in the analytic data. Fourth, potentially important confounders—including depression, stress, caffeine, medication, physical health, BMI, chronotype, and social support—were not measured, leaving open the possibility of residual confounding and limiting interpretation of the adjusted associations. Fifth, reliability and confirmatory-factor-analysis findings for the administered FSS-7 were specific to the present sample and did not constitute revalidation of the Arabic FSS-9.

The alternative conditional-ordering comparison does not resolve these limitations. It quantifies how the fitted serial product changes under two concurrent regression specifications, but it cannot determine whether fatigue precedes anxiety, anxiety precedes fatigue, or both reflect prior sleep or health processes.

The Harman and HTMT checks are limited descriptive diagnostics rather than proof that common-method variance is absent. The reliability and CFA findings provide sample-specific supportive evidence regarding the internal consistency and measurement structure of the instruments used in this study; they should not be interpreted as definitive validation of the administered FSS-7 form. Because this study was designed to examine associations rather than to conduct a standalone instrument-validation study, external validation, temporal data, and replication in probability-based samples remain necessary.

Gender emerged as an independent correlate of both anxiety and insomnia, yet it was modeled as a covariate rather than a moderator, so the present data cannot determine whether the relationship between anxiety and insomnia itself differs by gender. Addressing this question, together with establishing the order in which fatigue and anxiety unfold, will require future work that follows students over time or manipulates aspects of social media engagement directly.

## 6. Future Research Directions

Future studies should follow students across multiple time points to examine directionality and reciprocal associations. Clinical assessments aligned with DSM-5 criteria could complement symptom scales when the research addresses diagnostic questions. Combining problematic-use measures with prospective sleep records or device-based indicators, planning the measurement and adjustment of a wider range of potential confounders in advance based on theory and prior evidence, and recruiting through probability-based approaches across several Saudi universities would strengthen future evidence. Replication in other cultural settings would also help establish the generalizability of the findings.

## 7. Conclusions

Problematic social media use, fatigue, anxiety, and insomnia severity were concurrently associated in this sample of Saudi university students. Fatigue and anxiety symptoms statistically accounted for part of the conditional association between problematic social media use and insomnia severity, and the hypothesized fatigue-first serial indirect association was positive. Although the fatigue-first estimate was larger than the anxiety-first estimate, this difference did not establish temporal precedence. Daily social media-use duration showed a borderline association with insomnia severity that was not stable across sensitivity analyses, whereas female participants reported higher anxiety symptoms but lower insomnia severity than male participants in the adjusted models. The reported indirect associations and ordering contrast are descriptive cross-sectional results, not evidence of a temporal causal chain. Prospective and experimental studies with stronger confounder measurement are needed before making mechanism or intervention-sequence claims.

## Figures and Tables

**Table 1:** Descriptive statistics and intercorrelations among study variables (*N* = 430).

Variable	M	SD	Range	Skewness	Kurtosis	1	2	3
1. BSMAS	17.14	4.86	6–30	−0.02	0.01	—		
2. FSS	3.37	1.70	1–7	0.41	−0.86	0.462 **	—	
3. DASS-A	10.97	9.83	0–42	0.99	0.61	0.402 **	0.523 **	—
4. AIS	7.21	4.68	0–24	0.81	0.87	0.435 **	0.476 **	0.557 **

Note. BSMAS = Bergen Social Media Addiction Scale (6–30); FSS = Fatigue Severity Scale (1–7, mean); DASS-A = Depression Anxiety Stress Scales—Anxiety subscale × 2 (0–42); AIS = Athens Insomnia Scale (0–24). Gender and daily social media use were included as covariates in all regression analyses; therefore, they were not included in the Pearson correlation matrix. ** *p* < 0.01.

**Table 2:** Cross-sectional direct and indirect associations of problematic social media use with insomnia severity under hypothesized fatigue-first conditional ordering (*N* = 430).

Path	B	β	SE	t	*p*	95% CI
Outcome: Fatigue (FSS) R^2^ = 0.219, f^2^ = 0.28 (Medium)						
BSMAS → FSS (a_1_)	0.161	0.460	0.016	10.25	<0.001	[0.130, 0.191]
Gender	0.256	0.075	0.146	1.75	0.081	[−0.032, 0.543]
Daily social media use duration	0.012	0.007	0.075	0.153	0.878	[−0.137, 0.160]
Outcome: Anxiety symptoms (DASS-A) R^2^ = 0.318, f^2^ = 0.47 (Large)						
BSMAS → DASS-A (a_2_)	0.388	0.192	0.095	4.09	<0.001	[0.201, 0.574]
FSS → DASS-A (d_21_)	2.423	0.419	0.262	9.24	<0.001	[1.908, 2.939]
Gender	1.806	0.092	0.794	2.27	0.024	[0.244, 3.367]
Daily social media use duration	0.557	0.057	0.409	1.36	0.174	[−0.246, 1.360]
Outcome: Insomnia severity (AIS) R^2^ = 0.398, f^2^ = 0.66 (Large)						
BSMAS → AIS, direct (c′)	0.161	0.168	0.043	3.73	<0.001	[0.076, 0.247]
FSS → AIS (b_1_)	0.527	0.191	0.129	4.09	<0.001	[0.274, 0.780]
DASS-A → AIS (b_2_)	0.184	0.387	0.022	8.47	<0.001	[0.141, 0.227]
Gender	−0.859	−0.092	0.358	−2.40	0.017	[−1.563, −0.155]
Daily social media-use duration	0.362	0.078	0.184	1.97	0.049	[0.001, 0.723]
Total Effect						
BSMAS → AIS (c)	0.389	0.404	0.044	8.89	<0.001	[0.303, 0.475]
Indirect Effects (Bootstrap 5000 resamples)						
Path	B	—	BootSE	—	—	95% Boot CI
Ind1: BSMAS → FSS → AIS	0.085	—	0.024	—	—	[0.040, 0.135]
Ind2: BSMAS → DASS-A → AIS	0.071	—	0.018	—	—	[0.037, 0.108]
Ind3: BSMAS → FSS → DASS-A → AIS	0.072	—	0.016	—	—	[0.045, 0.105]
Total indirect effect	0.228	—	0.033	—	—	[0.166, 0.295]

Note. B = unstandardized coefficient; β = standardized coefficient; CI = confidence interval; Boot CI = bootstrapped confidence interval. Gender is coded as 1 = male, 2 = female. Daily social media use is coded as an ordinal frequency variable (1 = ≤1 h, 2 = 2–3 h, 3 = 4–5 h, 4 = 6–7 h, 5 = >7 h per day). Ind3 = serial indirect effect (BSMAS → FSS → DASS-A → AIS). *F*^2^ values of 0.02, 0.15, and 0.35 indicate small, medium, and large effects, respectively [47]. The shaded row indicates the primary hypothesized pathway and does not imply temporal precedence or causal mediation.

**Table 3:** Alternative cross-sectional conditional ordering (BSMAS → DASS-A → FSS → AIS; *N* = 430).

Path	B	SE	t	*p*	95% CI
BSMAS → DASS-A (a_1_)	0.777	0.093	8.37	<0.001	[0.594, 0.959]
BSMAS → FSS, controlling for DASS-A (a_2_)	0.107	0.015	6.93	<0.001	[0.077, 0.137]
DASS-A → FSS (d_21_)	0.069	0.007	9.24	<0.001	[0.054, 0.084]
DASS-A → AIS (b_1_)	0.184	0.022	8.47	<0.001	[0.141, 0.227]
FSS → AIS (b_2_)	0.527	0.129	4.09	<0.001	[0.274, 0.780]
BSMAS → AIS, direct (c′)	0.161	0.043	3.73	<0.001	[0.076, 0.247]
Indirect Effects (Bootstrap 5000 resamples)					
Ind1: BSMAS → DASS-A → AIS	0.143	0.026	—	—	[0.097, 0.197]
Ind2: BSMAS → FSS → AIS	0.056	0.018	—	—	[0.024, 0.094]
Ind3: BSMAS → DASS-A → FSS → AIS	0.028	0.009	—	—	[0.013, 0.046]
Total indirect effect	0.228	0.033	—	—	[0.163, 0.294]

Note. This alternative concurrent regression parameterization has the same outcome equation as the primary model; only the decomposition across the two mediator orderings differs. Unstandardized estimates are shown here. Bootstrap confidence intervals are used for indirect associations. The comparison does not test temporal precedence or causal mediation.

## Data Availability

The datasets are not publicly available because they are part of an ongoing research project, and additional analyses are being prepared for future publications. However, the data can be made available upon reasonable request from the corresponding author.

## Abbreviations

AIS, Athens Insomnia Scale; AVE, average variance extracted; BSMAS, Bergen Social Media Addiction Scale; CFI, comparative fit index; CI, confidence interval; DASS-21, Depression Anxiety Stress Scales-21; FSS-7, Fatigue Severity Scale, 7-item version; HTMT, heterotrait–monotrait ratio; RMSEA, root mean square error of approximation; SD, standard deviation; SE, standard error; SEM, structural equation modeling; SPSS, Statistical Package for the Social Sciences; SRMR, standardized root mean square residual; TLI, Tucker–Lewis index; VIF, variance inflation factor.
